# MicroRNA in Gastric Cancer Development: Mechanisms and Biomarkers

**DOI:** 10.3390/diagnostics10110891

**Published:** 2020-10-31

**Authors:** Fatimat Kipkeeva, Tatyana Muzaffarova, Alexandra Korotaeva, Maxim Nikulin, Kristina Grishina, Danzan Mansorunov, Pavel Apanovich, Alexander Karpukhin

**Affiliations:** 1 Research Centre for Medical Genetics, 1 Moskvorechye St., Moscow 115522, Russia; brca1@mail.ru (F.K.); tatyana.muzaffarova@mail.ru (T.M.); kor2306@mail.ru (A.K.); grstina@yandex.ru (K.G.); gah3ah@mail.ru (D.M.); apanovich2004@mail.ru (P.A.); 2 N.N. Blokhin National Medical Research Center of Oncology of the Ministry of Health of Russia, 24 Kashirskoe Shosse, Moscow 115478, Russia; maximpetrovich@mail.ru

**Keywords:** gastric cancer, microRNA, signaling pathways, metastasis, chemoresistance, markers

## Abstract

Gastric cancer (GC) is one of the most common and difficult diseases to treat. The study of signaling pathway regulation by microRNA provides information on the mechanisms of GC development and is the basis for biomarker creation. In this study, a circuit of microRNA interactions with signaling pathways was constructed. The microRNAs, associated with metastasis and chemoresistance, are described. In most cases, microRNAs in GC regulate the Wnt/β-catenin, PI3K/AKT/mTOR, RAS/RAF/ERK/MAPK, NF-kB, TGF-β, and JAK/STAT pathways. Part of the microRNA acts on several target genes that function in different pathways. This often leads to an intensification of the induced processes. MicroRNAs have also been described that have the opposite effect on different pathways, causing different functional consequences. By acting on several target genes, or genes associated with several pathways, microRNAs can function in a signaling network. MicroRNAs associated with metastasis most often interact with the Wnt/β-catenin pathway. MicroRNAs affecting chemoresistance, in most cases, affect the regulators of apoptosis and are associated with the PI3K/AKT/mTOR pathway. The characteristics of microRNAs proposed as candidates for GC biomarkers were analyzed. The currently developed diagnostic and prognostic panels of microRNAs are also considered.

## 1. Introduction

GC is the fourth most morbid and second most fatal cancer in the world and is a serious clinical problem due to late detection, resistance to chemotherapy, and poor prognosis [1]. Currently, new targets for the development of effective approaches to the treatment of GC, as well as informative diagnostic and prognostic biomarkers, are being actively pursued. In this regard, microRNAs are of great interest. MicroRNAs are small non-coding RNAs (18 to 24 nucleotides) that regulate many biological processes, including the development of cancer. MicroRNAs are regulators of signaling pathways, and thus are involved in various processes of a tumor cell, such as proliferation, invasion, migration, and metastasis. By interacting with certain genes in signaling pathways, microRNAs can suppress or induce the development of an oncological process. Thus, the study of the regulation of signaling pathways by using microRNAs provides information on the mechanisms of GC development and is the basis for the creation of diagnostic and prognostic markers. MicroRNAs, in addition to their functional properties, have structural features that ensure their attractiveness as markers—they are weakly subject to degradation in biological fluids, mainly due to their small size and movements within exosomes.

A number of works have been published in which the relationship of microRNAs with metastasis and tumor chemoresistance is considered, and potential GC biomarkers have been presented [2,3,4,5].

The interaction of microRNAs with signaling pathways has a number of features, which consist of different forms of regulation and are associated with the possibility of simultaneous action on several pathways. These features can lead to various functional and clinical consequences that do not follow directly from the action of microRNA on a specific gene. The possibility of microRNA action on several genes, as well as the possibility of genes functioning in several pathways and crosstalk between paths, have not been considered. Accordingly, these features are directly related to the choice of microRNAs for analysis as biomarkers or therapeutic targets. However, these features have not been previously summarized and analyzed. The relevance of a deeper analysis emphasizes the absence of effective and applied molecular systems for diagnostics and prognosis of GC [6]—a role microRNA is potentially capable of performing. It is also important not only to cite, but also to analyze the available data on potential microRNA-based biomarkers, in order to get a clearer picture of the current potential of this technology.

This review considers the functioning of microRNA in the format of a signaling network: the effect of microRNA on several target genes, as well as on genes associated with several pathways. MicroRNAs have been noted that have the opposite effect on different pathways and, as a result, cause different functional consequences. Information on microRNAs associated with metastasis and tumor resistance to GC chemotherapy in light of their interaction with signaling pathways is systematized. The characteristics of microRNA expression as candidates in GC biomarkers are critically analyzed. The microRNA panels developed to date are considered.

## 2. Features of the microRNA Interaction with Signaling Pathways in GC

**Figure 1:**
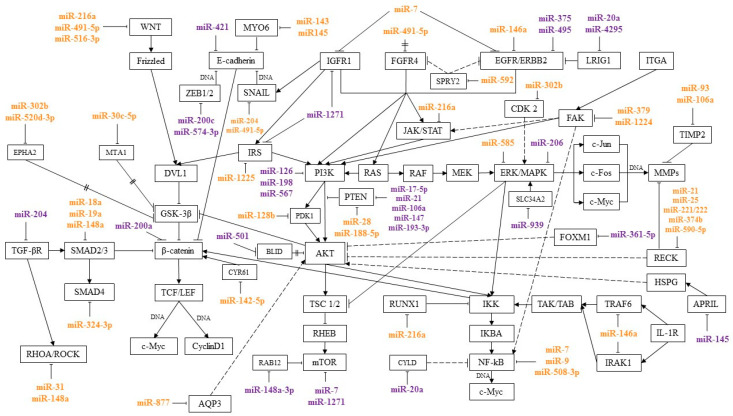
MicroRNAs and signaling pathways regulated by them in GC. miR—associated with metastasis; miR—associated with chemoresistance; 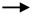 activation; 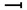 inhibition; 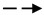 interaction is assumed; 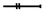 mediated interaction.

MicroRNAs are regulators of signaling pathways involved in various processes of a tumor cell, such as proliferation, invasion, migration, and colony formation. By interacting with certain genes in signaling pathways, microRNAs can suppress or induce cancer development. This review presents microRNAs involved in the pathogenesis of GC, and their relationship with signaling pathways was traced. Most of the works describe the relationship of microRNA with the Wnt/β-catenin, PI3K/AKT/mTOR, RAS/RAF/ERK/MAPK, NF-κB, TGF-β, and JAK/STAT pathways. These signaling pathways and the microRNAs regulating them are shown in Figure 1.

Activation of the Wnt/β-catenin signaling pathway is found in 10% to 50% of cases and in many GC cell lines. Wnt/β-catenin is one of the main signaling pathways involved in epithelial-mesenchymal transition (EMT) and plays a key role in metastasis. Suppression of the Wnt/β-catenin pathway inhibited the development of metastases in GC models [7,8].

PI3K/AKT/mTOR is involved in the regulation of the cell cycle and processes such as cell growth and metabolism. In a tumor cell, PI3K/AKT/mTOR is one of the main signaling pathways in the regulation of proliferation, invasion, and migration [9]. Protein kinase B (AKT) is one of the central proteins of the PI3K/AKT/mTOR pathway. Overexpression of p-AKT leads to dysregulation of the cell cycle, suppression of apoptosis, and activation of angiogenesis. In GC, increased levels of AKT and p-AKT expression were observed in 74% and 78% of cases. There was also a correlation between the level of p-AKT expression with the depth of tumor invasion and the degree of lymph node involvement [10]. AKT activation leads to phosphorylation of a number of proteins such as GSK3β, BAD, CASP9, Forkhead transcription factors, IκB kinase, and others. Proteins, depending on their type, are activated or inhibited as a result of phosphorylation. Glycogen synthase kinase 3 beta (GSK3β) is a regulator of the Wnt/β-catenin signaling pathway. Phosphorylation inhibits GSK-3, thereby activating the Wnt/β-catenin signaling pathway. AKT is also an activator of the NF-κB signaling pathway. IκB kinase (IKK) is a key component of the NF-κB pathway. Phosphorylation activates IKK and the NF-κB signaling pathway, inducing the expression of genes encoding antiapoptotic proteins [11,12]. In addition, in vitro and in vivo experiments have shown that activation of AKT/IκB/NF-κB induces EMT and leads to the development of metastases [13].

NF-κB is involved in the activation of the migratory and invasive properties of the cell, the regulation of apoptosis, and other cellular processes. In addition, this signaling pathway plays a key role in the regulation of the immune response and the inflammatory response. Activation of the NF-κB pathway not only promotes neoplastic cell transformation and the development of the oncological process, but also allows the tumor to avoid the response of the body’s immune system [14].

The RAS/RAF/ERK/MAPK signaling pathway interacts with PI3K/AKT/mTOR and activates NF-κB. MAPK is one of the key signaling pathways involved in the regulation of proliferative, migratory, and invasive cell properties and angiogenesis [15,16].

It was found that in GC the signaling pathways PI3K/AKT/mTOR, RAS/RAF/ERK/MAPK, and NF-kB can jointly participate in the regulation of a number of cellular processes and suppress the sensitivity of tumor cells to chemotherapy drugs—5-fluorouracil and cisplatin. In experiments on cell lines and tissue samples of GC, it was shown that PI3K/AKT/mTOR activates integrin-linked kinase (ILK), which, in turn, can activate the Ras/c-Raf/MEK1/2/ERK1/2/IκBα pathway and NF-κB. By activating the NF-κB signaling pathway, integrin-linked kinase induces EMT, angiogenesis, cell migration, and invasion, and suppresses apoptosis. ILK overexpression is associated with the depth of tumor invasion and the degree of metastasis to the lymph nodes in GC [17].

There is crosstalk between the NF-κB and JAK/STAT signaling pathways. The transcription factors NF-κB and STAT3 are activated by cytokines and regulate (sometimes jointly) the transcription of genes involved in apoptosis, proliferation, and other cellular processes. In GC, NF-κB and STAT3 act as oncogenes, enhancing the metastatic potential of tumor cells and contributing to the development and progression of the tumor [18].

The Ras homolog family member A/Rho-associated protein kinase 1 (RHOA/ROCK) and TGF-β pathways also induce the invasion and migration of tumor cells, and are associated with lymph node damage and EMT activation [19,20].

Thus, the relationship between the pathways described above forms a signaling network, leading to their joint participation in the regulation of cellular processes. When one pathway is activated, other signaling pathways associated with it can be activated, inducing a cascade of reactions leading, in particular, to suppression of apoptosis, and activation of invasive and migratory properties of a tumor cell.

MicroRNAs, acting on their target genes, can be inhibitors or activators of signaling pathways. For the most part, microRNAs, shown Figure 1, or their target genes interact with key effectors of these pathways, such as PI3K, AKT, PTEN, MAPK, mTOR, WNT, E-cadherin, β-catenin, IKK, and NF-κB.

MicroRNAs that inhibit several target genes, regulate several signaling pathways, and that can be involved in various cellular processes have been described. For example, miR-21 and miR-106 suppress the common target *PTEN*, as well as genes *RECK* and *TIMP2* that inhibit the metalloproteinases. This activates PI3K/AKT/mTOR and a number of signaling pathways with which metalloproteinases interact.

Some microRNAs can simultaneously be activators of one pathway and blockers of another. Thus, miR-216a blocks the JAK/STAT and Wnt/β-catenin pathways, inhibiting *JAK2* and *WNT3a*, and activates the NF-κB pathway, inhibiting the tumor suppressor gene *RUNX1*.

In addition, microRNAs regulate signaling pathways by inhibiting components of adjacent signaling cascades. In particular, miR-18a, miR19a, and miR-188-5p have been described as regulators of the Wnt/β-catenin signaling pathway. At the same time, miR-188-5p regulates this pathway, inhibiting *PTEN*, a key component of the PI3K/AKT/mTOR signaling pathway, and miR-18a and miR19a, inhibiting *SMAD2,* the TGF-β pathway mediator. Moreover, by inhibiting one target, microRNAs can act on two signaling pathways. These microRNAs include miR-592, which, by suppressing its target *SPRY2*, activated two signaling pathways, PI3K/AKT/mTOR and RAS/RAF/ERK/MAPK.

A more detailed consideration of the microRNA interaction with their targets and signaling pathways will make it possible to better understand the role of these microRNAs in the regulation of various cellular processes in GC. As mentioned above, a number of microRNAs act on several targets, regulating several signaling pathways. MiR-21 and miR-106a, by inhibiting *PTEN*, activate the PI3K/AKT/mTOR pathway, which leads to the induction of the proliferative properties of the tumor cell, as well as to the suppression of apoptosis and autophagy. In addition, the targets of miR-21 and miR-106a—*RECK* and *TIMP2*—are associated with the Wnt/β-catenin, PI3K/AKT/mTOR, RAS/RAF/ERK/MAPK, NF-κB, JAK/STAT, and Notch pathways. Suppression of *RECK* and *TIMP2* leads to activation of metalloproteinases and destruction of the extracellular matrix and basement membrane of cells, which are predictors of the metastatic process [21,22,23,24,25,26,27]. These microRNAs, by inhibiting various targets, affect a number of signaling pathways, which leads to the suppression of apoptosis and promotes the development of metastases.

MiR-302b is a suppressor of tumor growth and metastasis in GC. A decreased level of miR-302b expression is associated with the involvement of regional lymph nodes in the metastatic process, peritoneal carcinomatosis, and the development of distant metastases. Direct targets of miR-302b include *CDK2* and *EPHA2*. Transfection with miR-302b analogs inhibited the RAS/RAF/ERK/MAPK and Wnt/β-catenin signaling pathways in GC cells [28,29,30].

MiR-20a is a direct inhibitor of *LRIG1*. Suppression of *LRIG1* leads to the activation of EGFR and signaling pathways PI3K/AKT/mTOR and RAS/RAF/ERK/MAPK. This activates the ATP-binding cassette transporter P-gp (ABCB1), which causes an increase in the outflow of drugs from the cell. In addition, the anti-apoptotic protein BCL2 is also induced. The *CYLD* gene is another target of miR-20a. Suppression of *CYLD* activates the NF-κB signaling pathway and anti-apoptotic proteins Livin and Survivin.

Due to the action of these mechanisms, miR-20a overexpression caused an increase in the efflux of chemotherapy drugs from cultured GC cells, as well as apoptosis suppression, which led to the development of tumor cell chemoresistance [31,32].

Decreased miR-20a expression was shown to inhibit Wnt/β-catenin and RAS/RAF/ERK/MAPK signaling pathways. This, in turn, led to inhibition of growth, as well as the invasive and migratory properties of GC cells by in vitro experiments [33].

In experiments on GC cell lines, miR-491-5p suppressed cell migration and proliferation and promoted apoptosis. MiR-491-5p was originally described as an inhibitor of the antiapoptotic factor BCL-XL. MiR-491-5p has been shown to inhibit ERK1/2 and AKT. Later, it was found that direct targets of miR-491-5p are *SNAIL* and *WNT3A*. Overexpression of miR-491-5p inhibited FGFR4, N-cadherin, fibronectin (FN1), c-Myc, TCF-1, and CyclinD and activated E-cadherin, as a result of which miR-491-5p inhibited EMT [34,35]. Thus, miR-491-5p inhibits the signaling pathways PI3K/AKT/mTOR, Wnt/β-catenin, and RAS/RAF/ERK/MAPK, which leads to suppression of metastases development and induces apoptosis.

MiR-7 is a direct inhibitor of a number of targets—*mTOR, EGFR, IGF1R,* and *RELA*—and thus blocks several signaling pathways. By inhibiting its target *mTOR* (PI3K/AKT/mTOR pathway), miR-7 induces apoptosis and suppresses tumor growth in in vivo experiments [36]. As a result of suppression of its target RELA, miR-7 inhibits the NF-κB signaling pathway and genes associated with metastasis: *VNT, ICAM-1, VCAM-1, MMP-2, MMP-9*, and *VEGF* [37].

In Zhao et al., miR-7 was identified as a direct inhibitor of *IGF1R*, which is related to the IGF/IGF1R/IRS1 signaling pathway [38]. The products of the *EGFR, IGF1R, IRS1,* and *IRS2* genes, in addition to the regulation of cell proliferation, are involved in the mechanisms of cell migration. IGF1R is a predictor of neoplastic transformation and is involved in key stages of the metastatic cascade, such as adhesion, migration, invasion, colonization by tumor cells of distant organs, and angiogenesis. In experiments on GC cell lines, SNAIL was inhibited as a result of suppression of IGF1R, which led to the activation of E-cadherin and suppression of EMT [38]. Thus, by blocking the IGF/IGF1R/IRS1 signaling pathway, and others mentioned above, miR-7 is an inhibitor of EMT and metastatic processes in GC.

The genes *IGF1R, IRS1, mTOR*, and *BCL2* have been identified as direct targets of miR-1271 in GC [39]. *IGF1R* and *IRS1* belong to the IGF/IGF1R/IRS1 signaling pathway, which is often considered as part of the PI3K/AKT/mTOR pathway and is involved in the regulation of cell proliferation and apoptosis [40]. IGF/IGF1R/IRS1 is an activator of the PI3K/AKT/mTOR pathway. As a result of PI3K/AKT/mTOR activation, the antiapoptotic protein BCL2 is induced [41]. Thus, by acting on a number of targets, miR-1271 inhibits cell proliferation and induces apoptosis [39].

Despite the fact that both miR-7 and miR-1271 are inhibitors of the same signaling pathway (IGF/IGF1R/IRS1), they act as regulators of different cellular processes: miR-7 is described as an EMT inhibitor, and miR-1271 as an inducer apoptosis. Thus, even if microRNAs initially act on the same signaling pathway, they can regulate different cellular processes.

Some microRNAs are capable of multidirectional effects on signaling pathways. In particular, miR-216a and miR-148a have been described as inhibitors of one signaling pathway and activators of another. MiR-216a inhibits the Wnt/β-catenin and JAK/STAT signaling pathways by suppressing the direct targets *Wnt3a* and *JAK2*. In Tao et al., overexpression of miR-216a led to the activation of E-cadherin and inhibited EMT in in vitro experiments. The decreased level of miR-216a expression correlated with metastasis to the lymph nodes in patients with GC [42,43]. At the same time, miR-216a is an inducer of the NF-κB signaling pathway. Overexpression of miR-216a activated the NF-κB signaling pathway by suppressing *RUNX1* and led to an increase in the expression levels of CyclinD1, BCL2, MMP-2, and MMP-9, inducing the migratory, proliferative, and invasive properties of cells [44]. Therefore, miR-216a plays a dual role in the progression of GC. On the one hand, this microRNA inhibits the Wnt/β-catenin and JAK/STAT signaling pathways, acting as a tumor suppressor and preventing metastasis; on the other hand, it promotes the development of metastases by activating the NF-κB pathway.

MiR-148a inhibits the TGF-β and RHOA/ROCK pathways, suppressing the targets of *SMAD2* and *ROCK1*. The TGF-β signaling pathway is one of the regulators of EMT. TGF-β activation correlates with an increase in the metastatic potential of GC cells. MiR-148a, by inhibiting *SMAD2* and the TGF-β pathway, led to the activation of E-cadherin and suppression of WNT. This caused suppression of EMT and reduced the ability of GC cells to invade and migrate [45]. In addition, in experiments on GC cells, miR-148a inhibited ROCK1-induced invasion and migration of GC cells [46]. At the same time, miR-148a activates m-TORC1 (PI3K/AKT/mTOR pathway), inhibiting *RAB12*. The direct target of miR-148a, *RAB12*, is a potent autophagy inducer. Autophagy is the degradation of cell organelles and proteins used as a source of additional energy. This allows the tumor cell to survive in adverse conditions. In in vitro experiments, miR-148a inhibited *RAB12* and, as a consequence, autophagy. As a result, overexpression of miR-148a increased the sensitivity of tumor cells to cisplatin [47].

Therefore, by exerting an opposite effect on different pathways—inhibiting the TGF-β and RHOA/ROCK pathways, and activating PI3K/AKT/mTOR—miR-148a causes various functional consequences: It suppresses the development of metastases and increases the sensitivity of tumor cells to chemotherapy.

As noted above, a number of microRNAs regulate signaling pathways by interacting with mediators of adjacent signaling cascades. Thus, miR-188-5p inhibits PTEN, while AKT is activated. The AKT, in addition to the PI3K/AKT/mTOR pathway, activates the Wnt/β-catenin pathway, suppressing GSK3β. In cancer cell lines, overexpression of miR-188-5p inhibited PTEN and GSK3β, resulting in an increase in the expression levels of gene-effectors of the Wnt/β-catenin signaling pathway. MiR-188-5p enhanced the cells’ ability to migrate and invade and their metastatic potential [48].

MiR-18a, miR19a, and miR-324-3p act on components of the TGF-β pathway and activate the Wnt/β-catenin pathway. *SMAD2*, a direct target of miR-18a and miR19a, is associated with the TGF-β pathway. Moreover, SMAD2 is an inhibitor of β-catenin (Wnt/β-catenin pathway). It was experimentally shown that miR-18a and miR-19b, by inhibiting *SMAD2*, increased the expression levels of β-catenin, C-Myc, and axin2, activated the Wnt/β-catenin signaling pathway and the metastatic potential of GC cells. Induced expression of miR-18a/19a significantly enhanced the ability of GC cells to migrate and proliferate [49]. The direct target of miR-324-3p is *SMAD4*. In in vitro experiments, miR-324-3p inhibited apoptosis and promoted proliferation, migration, and survival of GC cells [50].

The *APRIL* gene has been identified as a direct target of miR-145 in GC. In experiments on cell lines, it was shown that APRIL, interacting with Heparan Sulfate Proteoglycan (HSPG), phosphorylates AKT. This, in turn, activates the NF-κB signaling pathway and the effectors of this pathway—BCL2 and BCL-XL (inhibitors of apoptosis). Suppression of APRIL led to inactivation of AKT and the NF-κB signaling pathway, and promoted apoptosis [51].

By suppressing its direct target *PDK1*, miR-128b also affected AKT, while inhibiting the NF-κB pathway in GC. It was shown that miR-128b inhibited p-AKT and NF-κB in GC cells and inactivated the PDK1/AKT/NF-κB axis [52].

MiR-361-5p is a *FOXM1* inhibitor that affects the PI3K/AKT/mTOR pathway. The FOXM1 transcription factor belongs to the Forkhead family. According to Tian et al., FOXM1 inhibits the PI3K/AKT/mTOR pathway. In vitro experiments miR-361-5p inhibited *FOXM1* and led to an increase of p-AKT and mTOR. As a result of activation of the PI3K/AKT/mTOR signaling pathway, miR-361-5p inhibited autophagy and increased the sensitivity of tumor cells to chemotherapy [53].

The direct target of miR-582, *FOXO3,* also belongs to the Forkhead family. As a result of *FOXO3* suppression, miR-582 induced the PI3K/AKT/mTOR signaling pathway. This, in turn, led to the activation of the transcription factor SNAIL, which is an inhibitor of E-cadherin and is associated with the Wnt/β-catenin pathway [54]. Thus, by activating the PI3K/AKT/SNAIL signaling pathway, miR-582 led to the induction of EMT, which promoted the growth, invasion, and GC metastasis [55].

The direct target of miR-877, aquaporin 3 (*AQP3*), is also a regulator of the PI3K/AKT/mTOR pathway. By activating the PI3K/AKT/SNAIL axis, AQP3 induces EMT. In addition, AQP3 has been shown to enhance the resistance of GC cells to cisplatin. In vitro experiments, overexpression of miR-877 inhibited AQP3, resulting in suppression of EMT, invasion and proliferation of GC cells, and also induced apoptosis [56].

Thus, by inhibiting genes for some signaling pathways, miR-18a, miR-19a, miR-128b, miR-145, miR-188-5p, miR-324, miR-361-5p, miR-582, and miR-877 acted on other cascades connected with these paths.

By inhibiting one target, microRNA regulated two signaling pathways. In particular, it was shown that miR-592, as a result of suppression of its target *SPRY2*, activated two signaling pathways, PI3K/AKT/mTOR and RAS/RAF/ERK/MAPK. This led to the induction of the proliferative, invasive, and migratory properties of GC cells [57]. MiR-1224, being a direct inhibitor of focal adhesion kinase (*FAK*), inhibits the activity of the JAK/STAT and NF-κB signaling pathways and inhibits EMT. In addition, in experiments on GC cell lines, a decrease in miR-1224 expression led to an increase in *WNT* and *ZEB1* expression levels, a decrease in E-cadherin levels, and induced cell migration. It was shown in vivo that miR-1224 prevents the development of distant metastases [58]. By influencing different genes and pathways, microRNAs may be able to exert a unidirectional effect on cellular processes.

Thus, by acting on several target genes or genes associated with several pathways, microRNAs can function in the format of a signaling network. It is still impossible to say with certainty on how many pathways a particular microRNA affects and to what extent. However, the signaling pathways in which the greatest amount of microRNA is involved in regulation during GC are indicated. In most cases, according to the available data, microRNAs regulate the Wnt/β-catenin, PI3K/AKT/mTOR, RAS/RAF/EKK/MAPK, NF-kB, TGF-β, and JAK/STAT pathways. As follows from the data in Figure 1, a significant proportion of microRNAs associated with metastasis regulate the Wnt/β-catenin pathway. The largest amount of microRNAs associated with GC chemoresistance is involved in the regulation of the PI3K/AKT/mTOR pathway.

## 3. MicroRNAs Associated with GC Metastasis and Signaling Pathways Regulated by Them

The development of metastases is one of the reasons for the high mortality rate in cancer, including GC. By regulating signaling pathways, microRNAs play an important role in the process of metastasis. This section examines microRNAs, the expression level of which changes with the development of the metastatic processes and shows their association with the development of metastases (with metastasis to the lymph nodes and with the development of distant metastases).

MicroRNAs, considered in the experimental works in connection with metastasis of GC, regulate the Wnt/β-catenin pathway. Activation of this path leads to the launch of EMT. MicroRNA targets are either the genes of the Wnt/β-catenin pathway (for example, *WNT1*), or genes associated with this pathway, for example *MTA, MYO6,* and *IRS1.* The products of these genes regulate the Wnt/β-catenin pathway: MTA is an inhibitor of GSK3β [59], MYO6 inhibits E-cadherin [60], and IRS1 activates DVL2 [61].

The expression of WNT (a ligand of the Wnt/β-catenin pathway) is influenced by miR-216a, miR-491-5p, and miR-516a-3p. Suppression of these microRNAs leads to an increase in WNT expression and subsequent activation of the Wnt/β-catenin pathway, which stimulates the development of metastases [35,43,62].

MiR-143, miR-145, miR-204, and miR-491-5p activate E-cadherin (Wnt/β-catenin pathway) by suppressing direct targets: *MYO6* (miR-143 and miR-145) and *SNAIL* (miR-204 and miR-491-5p), thereby inhibiting EMT and metastasis. [34,60,63].

Overexpression of miR-93 and miR-106a is associated with metastasis to the lymph nodes and invasion into the vascular bed during GC. TIMP2, which is an E-cadherin inducer and an EMT inhibitor, was identified as a direct target of these microRNAs. In addition, TIMP2 has been shown to inhibit matrix metalloproteinases, which degrade the extracellular matrix and basement membrane, which is a prerequisite for metastasis. Suppressing *TIMP2*, miR-93, and miR-106a contributes to the development of metastases [27,64]. However, data on the role of TIMP2 in the GC development and metastasis are ambiguous; there is information about the relationship between TIMP2 overexpression and GC progression and the distant metastases development [65].

GSK3β is a major component of the Wnt/β-catenin pathway. GSK3β is indirectly affected by miR-30c-5p, miR188-5p, miR-302b, and miR-520d-3p. MiR-30c and miR-302b activate GSK3β, inhibiting their targets *MTA1* and *EPHA2*. Activation of GSK3β leads to inhibition of the Wnt/β-catenin pathway. Thus, miR-30c and miR-302b suppress the development of metastases. MiR188-5p, on the contrary, enhances the metastatic potential of GC cells. Its immediate target is *PTEN*. PTEN protein is an AKT inhibitor. AKT inhibits GSK3β and activates the Wnt/β-catenin pathway. Thus, miR-188-5p, by blocking PTEN, activates the Wnt/β-catenin signaling pathway, which stimulates EMT and metastasis [30,48,59,66].

A number of microRNAs regulate the PI3K/AKT/mTOR signaling pathway. Basically, microRNAs act on the components of this pathway indirectly. For example, miR-340, miR-379, miR-520a-3p, and miR-1254 inhibit the PI3K/AKT/mTOR signaling pathway by suppressing the direct targets *SPP1, FAK, WEE1*, and *SMURF1*. It is reported that an increase in the expression levels of miR-379, 520a-3p, 1254, and miR-340 resulted in a decrease in AKT phosphorylation [67,68,69,70]

The other microRNAs directly interact with the main components of the PI3K/AKT/mTOR pathway. *PI3K* is acted upon by miR-142-5p, miR-216, and miR-491, inhibiting this pathway. MiR-28 and miR-197 are regulators of *PTEN*, an inhibitor of the PI3K/AKT/mTOR pathway. But the action of these two microRNAs is opposite. MiR-28, by inhibiting PTEN, activates the PI3K/AKT/mTOR signaling pathway [71]. MiR-197 is a direct metadherin (*MTDH*) inhibitor, which is a PTEN blocker. Thus, by suppressing *MTDH*, miR-197 activates PTEN and thereby inhibits the PI3K/AKT/mTOR signaling pathway [72].

By regulating the PI3K/AKT/mTOR cascade, microRNAs are involved in the processes of invasion, migration, and EMT. As noted in other works [67,68,70,73], microRNAs influencing the PI3K/AKT/mTOR pathway also affected the genes associated with EMT (*E-cadherin*, *N-cadherin*, and *WNT*).

MiR-7, miR-9, and miR-508-3p act as tumor suppressors by inhibiting the NF-κB pathway [37,74,75].

Information regarding miR-216a is contradictory. According to Wu et al., it is an oncogene, an activator of the NF-κB pathway. The increased expression of miR-216a correlated with lymph nodes metastasis [44]. On the other hand, according to Tao et al. and Song et al., miR-216a is a tumor suppressor. It suppresses JAK2 (JAK/STAT pathway) and prevents the metastases development [42].

MiR-34a and miR-146a belong to the inhibitors of EMT, which was identified as a key factor in the process of metastasis. EMT-inducing signaling pathways such as Wnt/β-catenin, TGF-β, and NF-κB are common targets for miR-34a and miR-146a [76,77,78]. In our recent work, it was shown that miR-34a and miR-146a are associated with GC metastasis to regional lymph nodes and with the distant metastases development. Expression levels of miR-34a and miR-146a decreased with regional lymph nodes involvement in the metastatic process and continued to decrease with the distant metastases development [79].

Interestingly, miR-21, miR-25, miR-221/222, miR-374b-5p, and miR-590-5p, by suppressing their *RECK* target, activate several signaling pathways. RECK is a potent inhibitor of metalloproteinases that inhibits the AKT/ERK, JAK/STAT, and Notch signaling pathways. The *RECK* gene is a tumor suppressor and prevents the metastases development. In GC, there is an increased level of expression of microRNAs that inhibit *RECK*. Accordingly, overexpression of these microRNAs promotes lymph nodes metastasis [24,80,81,82,83].

Table 1 shows microRNAs role in the metastasis process, their direct target genes, and signaling pathways regulated by them. Statistical significance for the microRNAs association with metastasis was *p* < 0.05. In most studies, the works were carried out both on GC samples (GC tissue and paired adjacent normal tissues) and on cell lines and xenografts using reverse transcription polymerase chain reaction (RT-PCR). Statistical methods and significance of the results for the microRNAs which may be considered as candidates in biomarkers are shown in Table 3. In most cases (as presented in Figure 1 and Table 1), microRNAs associated with metastasis regulate Wnt/β-catenin pathway.

Thus, impaired expression of a number of microRNAs is associated with the development of a metastatic process. In most cases, the studied microRNAs act as tumor suppressors, and their reduced expression is associated with the metastases development.

In a greater number of cases, the microRNAs discussed in this section affect the Wnt/β-catenin pathway, a key regulator of EMT. In addition, many authors have shown that microRNAs that regulate other signaling pathways (in particular, PI3K/AKT/mTOR, NF-κB, and JAK/STAT) also affect the expression of E-cadherin, N-cadherin, and WNT [58,67,68,70,73].

## 4. MicroRNAs Associated with the Development of GC Chemoresistance and Signaling Pathways Regulated by Them

Tumor resistance to chemotherapy leads to an unfavorable outcome of the disease and is an urgent and complex problem in the treatment of cancer, including GC. Recently, the mechanisms of tumor chemoresistance development have been actively studied [96]. Tumor resistance to chemotherapy, among other factors, is associated with a change in the microRNA expression and, as a consequence, with deregulation of cell signaling pathways. PI3K/AKT/mTOR is one of the key pathways involved in the tumor chemoresistance development [97]. This pathway interacts with the largest number of microRNAs associated with the development of resistance to chemotherapy. In addition to influencing proliferation, the PI3K/AKT/mTOR pathway is associated with processes such as apoptosis and autophagy. Most often, microRNAs act as apoptosis regulators, and less often act on ATP-binding cassette transporters, which are responsible for the outflow of drugs (toxins) from the cell. In several cases, microRNAs involved in autophagy have been described (Table 2).

MicroRNAs can both enhance and suppress chemotherapy-induced apoptosis. Many studies have shown the relationship between changes in the microRNAs expression and proteins that regulate apoptosis. MiR-501 and miR-4295 suppress apoptosis and the sensitivity of GC cells to chemotherapy. These microRNAs, by inhibiting the direct targets *BLID* and *LRIG1*, activate the PI3K/AKT/mTOR pathway. As a result, the antiapoptotic factor BCL2 is induced and the proapoptotic proteins BAX, CASP3, and CASP9 are inhibited [96,98].

Inhibitors of apoptosis also include miR-17-5p, miR-21, miR-106a, miR-147, and miR-193-3p. Their direct target is *PTEN*, a suppressor of the PI3K/AKT/mTOR signaling pathway. By inhibiting PTEN, these microRNAs activate the PI3K/AKT/mTOR pathway and suppress apoptosis. A decrease in the expression of miR-17-5p and miR-147 inhibited proliferation, induced apoptosis, and increased the sensitivity of GC cells to chemotherapy [99,100]. A decrease in miR-193-3p expression inhibited proliferation and migration, and also increased the sensitivity of GC cells to 5-Fluorouracil (5-FU) in vitro and tumor growth in vivo [101].

In opposite, miR-198, miR-375, and miR-495 are inducers of apoptosis. Their overexpression increased the sensitivity of GC cells to chemotherapy. The PIK3R1 gene was identified as a direct target for miR-198, and ERBB2 for miR-375 and miR-495. By suppressing their targets, miR-198, miR-375, and miR-495 inhibited the PI3K/AKT/mTOR signaling pathway. The inhibition of miR-198, miR-375, and miR-495 led to the development of resistance of tumor cells to cisplatin [102,103,104].

Some apoptosis-associated microRNAs are involved in other signaling pathways: RAS/RAF/ERK/MAPK, Wnt/β-catenin, and NF-κB.

MiR-206 and miR-939 regulate the RAS/RAF/ERK/MAPK pathway. MiR-206 is a direct inhibitor of *MAPK3*, and miR-939 inhibits *SL3C4A2* (SL3C4A2/Raf/MEK/ERK pathway). Transfection with miR-206 suppressed the ability of GC cells to proliferate, invade, and migrate, and induced apoptosis. In experiments on GC cell lines, overexpression of miR-939 led to an increase in the level of CASP3 expression and, consequently, to an increase in the efficiency of 5-FU-induced apoptosis [105,106].

MiR-135b is also a regulator of the RAS/RAF/ERK/MAPK pathway, but the information on this microRNA is contradictory. According to Zhou et al., miR-135b, by inhibiting its target *MST1*, activated the RAS/RAF/ERK/MAPK pathway. The blocking of miR-135b led to the induction of apoptosis and suppression of GC cell proliferation, which increased their sensitivity to cisplatin [107]. However, according to Wang et al., overexpression of miR-135b induced apoptosis of GC cells in vitro and increased tumor sensitivity to chemotherapy in vivo. The authors showed that miR-135b inhibited the RAS/RAF/ERK/MAPK pathway, suppressing its target *ITGA2*. As a result, miR-135b suppressed the anti-apoptotic protein BCL2 and induced the expression of the pro-apoptotic protein BAX [108].

MiR-421 target genes *E-cadherin* and *CASP3* are key regulators of EMT and apoptosis. Overexpression of miR-421 increased the metastatic potential of GC cells, inhibited apoptosis, and induced chemoresistance. Transfection of miR-421 inhibited the activity of *CASP3*, resulting in inhibition of apoptosis in GC cells treated with cisplatin [109].

MiR-200c, miR-204, and miR-574-3p have the opposite effect. They suppress EMT and induce apoptosis of GC cells. In experiments in vitro, miR-200c and miR-574-3p activated *E-cadherin* by suppressing direct targets—*ZEB1* and *ZEB2*. As was shown, knockdown of *ZEB2* due to overexpression of miR-200c activated *BAX* and *CASP3* and decreased the level of *BCL2* expression. CASP3 is also activated by miR-204 [110,111,112]. The influence of miR-200a on EMT is realized via inhibiting β-catenin and, consequently, the Wnt/β-catenin signaling pathway [113].

MiR-146a and miR-362-5p enhance apoptosis via regulation of the NF-κB signaling pathway. Overexpression of miR-146a led to the activation of CASP3, suppression of BCL2, and induction of GC cell apoptosis as a result [114]. MiR-362-5p is a direct inhibitor of *SUZ12*. Knockdown of *SUZ12* increased the sensitivity to cisplatin and decreased the levels of the NF-κB/p65 protein, as well as enhanced cisplatin-induced apoptosis in GC cells [115].

MiR-34c suppressed BCL2 and activated CASP9 and BAX. Overexpression of miR-34c increased the sensitivity of GC cells to chemotherapy, induced apoptosis, and inhibited proliferation [116]. Interestingly, although miR-524-5p inactivated CASP3, overexpression of miR-524-5p significantly reduced the ability of GC cells to proliferate and promoted apoptosis [117].

The effect of miR-218 on the apoptosis inhibitor Survivin can increase the sensitivity of tumor cells to chemotherapy. Transfection of this microRNA inhibited the expression of Survivin in GC cells, which promoted apoptosis and increased the sensitivity of cancer cells to cisplatin [118].

The microRNAs that, in addition to apoptosis, affect the activity of multidrug resistance (MDR) proteins are also described. MiR-20a, already considered in the first section, activates the PI3K/Akt, MAPK/ERK, and NF-kB signaling pathways. As a result, the ATP-binding cassette transporter ABCB1, and the antiapoptotic proteins BCL2, Livin, and Survivin are induced, which leads to the development of chemoresistance of tumor cells [31,32].

MiR-567 inhibits its target *PIK3AP1* and the PI3K/AKT/c-Myc pathway. The c-Myc induces drug resistance by regulating the expression of ATP-binding cassette transporters. MiR-567 increases the sensitivity of GC cells to cisplatin and 5-FU, inhibits the proliferative properties of cells in vitro, and slows down tumor growth in vivo. In experiments on cell lines, GC miR-567 increased the expression levels of *CASP3* and *CASP9*, inducing cell apoptosis [119].

The long non-coding RNA HOTAIR is a direct inhibitor of miR-34a and miR-126. HOTAIR knockdown induced the expression of miR-34a and miR-126. In vitro experiments showed that inhibition of HOTAIR inhibited the expression of ATP-binding cassette transporters, which increase the efflux of toxins from the cell and are associated with multidrug resistance mediated by the ABCB1, ABCC1, and ABCG2, MRP1 genes. Suppression of HOTAIR activated miR-34a. As a result, the expression levels of the pro-apoptotic proteins CASP3 and BAX were increased, while the expression levels of the apoptosis inhibitors BCL2 and Survivin were decreased. In addition, levels of WNT1 and β-catenin expression were inhibited [120]. Overexpression of HOTAIR and the corresponding inhibition of miR-126 led to an increase in the proliferative properties of cells and inhibition of apoptosis in vitro. In experiments on GC cell lines, inhibition of miR-126 led to the activation of its direct targets *VEGFA* and *PIK3R2*, the PI3K/AKT signaling pathway, and was associated with the drug resistance *MRP1* gene [121].

MiR-148a, miR-361-5p, and miR-30e have been characterized as inhibitors of autophagy. MiR-148a and miR-361-5p activate the PI3K/AKT/mTOR pathway, inhibiting their targets *RAB12* and *FOXM1*. The overexpression of miR-148a and miR-361-5p increased the sensitivity of tumor cells to chemotherapy drugs [47,53].

MiR-30e is regulated by the long non-coding RNA MALAT1. It was shown that suppression of MALAT1 leads to overexpression of miR-30e, inhibition of autophagy, and increased sensitivity of GC cells to cisplatin [122].

The drug resistance of tumor cells is closely related to the main signs of malignant transformation. Many microRNAs that affect chemoresistance are also involved in cellular processes such as invasion, migration, and proliferation, and are important for the metastasis process.

In many articles on the study of microRNAs associated with chemoresistance, it is assumed that the suppression of microRNAs—inhibitors of apoptosis—can be an effective therapeutic strategy. In this regard, it should be taken into account that microRNAs can act on more than one target (signaling pathway), regulating other processes in addition to apoptosis. Some microRNAs can have a multidirectional effect on cellular processes. Thus, when analyzing microRNAs capable of inhibiting apoptosis, one should take into account their ability to exert multiple influences, including those described in this work.

**Figure 2:**
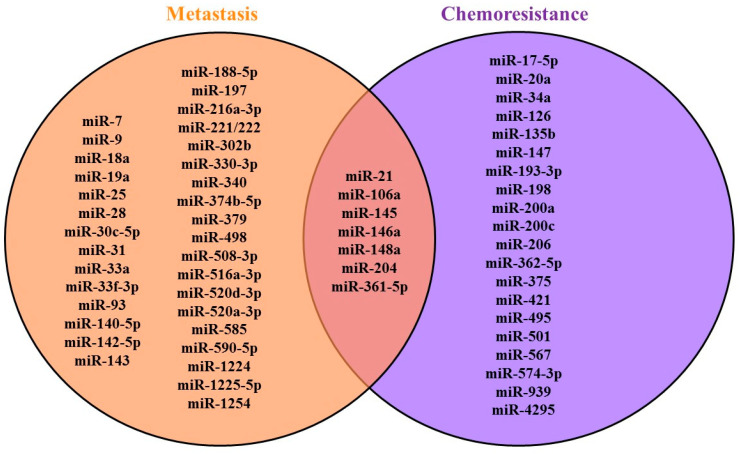
MicroRNAs in GC metastasis and chemoresistance.

Several microRNAs associated with chemoresistance are also involved in invasion and migration; thus, they are also important for metastasis [83,100,101,105,106,109,110,111,112,113,120]. Some of microRNAs were involved in both metastasis and chemoresistance, as shown in Figure 2.

## 5. MicroRNA as GC Prognostic and Diagnostic Markers

MicroRNAs can act as convenient clinical biomarkers, given their high stability in different body environments and differential expression in GC. Circulating and tissue microRNAs can allow detection of GC at an early stage, assessment of the course of the disease in dynamics, including determining the likelihood of relapse and (or) metastasis, and prediction of the tumor response to chemotherapy. Thus, it is expected that the use of microRNAs as markers will increase the sensitivity and specificity of diagnostic and prognostic tests for GC [123].

Currently, numerous studies are being carried out, including microarrays, to identify differentially expressed microRNAs in GC. In many articles where the relationship of microRNAs with signaling pathways was considered, the authors report the possibility of using various microRNAs as prognostic or diagnostic markers. In these studies, it was shown that the expression level of the investigated microRNAs significantly differed in the GC tissue, as compared to the unaffected mucosa, and was associated with metastasis. For example, Guan et al. found that miR-93 overexpression was correlated with lymph node metastasis in GC (*p* < 0.01) [64]. Cha et al. reported that decreased miR-140-5p expression correlates with lymph node metastasis (*p* = 0.018) and is further associated with a poor prognosis (progression free survival (PFS) and overall survival (OS), *p* < 0.05) [85]. 

Table 3 shows the microRNAs characteristics considered as candidates of GC biomarkers. In addition to microRNAs with known interactions with signaling pathways, this section also includes microRNAs for which such a relationship was not considered.

The microRNAs listed in Table 3 have different levels of evidence for their acceptability as markers. The closest to practical use are marker panels. However, data on a number of individual microRNAs demonstrate their significant potential as markers. These microRNAs, presented in Table 3, include miR-101-3p, miR-106a, miR-135, miR-140-5p, miR-196, and miR-552, which have a significant relationship with the clinical characteristics under study (given in Table 3). MiR-421, when associated with a number of clinical features, is most significantly associated with the early stage of GC, which indicates its promising potential as a diagnostic marker. It is also worth highlighting miR-129, the expression of which in GC differs from its expression among patients with benign gastric diseases.

At the same time, not all microRNAs positioned as candidates for GC markers have been studied from the point of view of possible expression changes in other gastric diseases. In particular, microRNA regulation can be impaired not only in malignant tumors but also under *Helicobacter pylori* infection, chronic gastritis, atrophic gastritis, intestinal metaplasia, and early dysplasia [150]. For example, according to Link et al., expression levels of miR-155 and miR-223 were increased in both GC and atrophic gastritis. No statistically significant differences in the expression levels of miR-155 and miR-223 were found between GC samples and atrophic gastritis [132]. Some microRNAs associated with GC, for example, miR-16, miR-17-5p, miR-20a, miR-22, miR-126, miR-132, miR-143, miR-191, miR-195, and miR-200, are also considered as markers of other (including frequent) diseases, such as cerebral atherosclerosis, endometriosis, and heart disease [151,152,153,154].

In addition, the results of published studies on some microRNAs are inconsistent. MiR-216a, according to Tao et al. [42], suppresses the metastases development. On the other hand, Wu et al. report that miR-216a promoted the metastases development and was associated with a poor prognosis [44]. MiR-135b, according to different authors, is considered both an apoptosis inhibitor and an inducer [107,108]. As shown by the results of the meta-analysis, the value of miR-200c as a diagnostic and prognostic marker cannot be considered definitively determined [136].

The circulating microRNAs constitute a significant part of microRNAs investigated as potential markers. They have generated significant interest, since their definition refers to minimally invasive diagnostic methods. Many of the circulating microRNAs have high sensitivity, specificity AUC, RR, etc. (Table 3). However, there are issues with circulating microRNA that require clarification. In particular, when comparing the expression levels of miR-196a and miR-196b in the blood serum and tumor tissues of the stomach, the correlation coefficient was 0.53 and 0.45. That is, the expression level of circulating microRNA in a significant number of cases did not correspond to the expression levels of tissue miR-196a and miR-196b [135].

In order to increase the sensitivity and specificity of microRNAs as markers, panels are being developed that include several microRNAs. One of the earliest panels was proposed back in 2011. Liu et al. developed a panel of five serum microRNAs (miR-1, miR-20a, miR-27a, miR-34, and miR-423-5p) for GC detection. For this panel, AUC = 0.879. This was higher than for other biomarkers, including CEA (AUC = 0.503) and CA19-9 (AUC = 0.6) [155]. A panel of five serum microRNAs (miR-16, miR-25, miR-92a, miR-451, and miR-486-5p) was proposed by Zhu et al. The expression level of these microRNAs was increased in GC patients compared to the control group. ROC (receiver operating characteristic) analysis showed high diagnostic accuracy for the early stage of GC [156]. Jiang et al. examined a panel of four microRNAs (miR-143-3p, miR-146a, miR-451a, and miR-501-3p) as non-invasive biomarkers to predict the lymph node metastases development in GC. The experimental group included 279 people; the validation group included 180 people. The characteristics of this panel in the validation group were AUC = 0.822 (95% CI, 0.758 to 0.875), specificity = 87.78%, and sensitivity = 63.33%. Moreover, Kaplan–Meier analysis showed that patients with lymph-node metastases and low levels of miR-146a and miR-451a expression had the worst OS (*p* < 0.05) [147].

Expression databases are often used in panel design. Zhao et al. used clinical and microRNA-seq data from patients with gastric adenocarcinoma (*n* = 310) downloaded from The Cancer Genome Atlas (TCGA) database. A microRNA prognostic panel (hsa-mir-7-2, hsa-mir-9-3, hsa-mir-548o, hsa-mir-1255a, and hsa-mir-3687) was developed for GC patients, but panel validation by RT-PCR was not performed. For validation, a verification group was formed using patient data from the TCGA database. In the experimental group, the AUC was 0.939, in the verification—0.901. The sensitivity and specificity of the panel were not indicated in the paper text. From the presented graphs of the ROC analysis, the sensitivity was about 90% for both groups, the specificity is about 75%. The Kaplan–Meier log rank test showed that low-risk patients had significantly longer survival times than high-risk patients (HR, 2.840; 95% CI, 1.937–4.162; *p* < 0.01). An appropriate formula was developed to use this panel. However, the authors have shown that a history of relapse and age over 65 years are independent prognostic factors [146].

In 6% to 16% of cases, gastric adenocarcinoma is accompanied by infection with the Epstein–Barr virus (EBV), which expresses microRNAs in the tumor [157]. Treece et al. developed the GastroGenus miR panel, which includes microRNAs encoded by the Epstein–Barr virus (EBV) and cancer-specific microRNAs. In GC tissues infected with EBV, an increased expression of EBV-encoded microRNAs (*p* < 0.006) was compared with uninfected ones. Concomitant dysregulation of four hsa-miRs expression (*p* < 0.00125) was observed with overexpression of EBV-microRNA. There was formed a hypothesis that EBV infection could affect the regulation of GC cell signaling pathways [158]. It is assumed that this panel will differentiate EBV-positive GC from EBV-negative one.

The number of microRNAs studied in connection with the clinical manifestations of GC is steadily increasing. Diagnostic and prognostic microRNAs and their panels are being actively developed. However, to date, no microRNA (microRNA panel) has been found that could be used in clinical practice.

In the European Society for Medical Oncology (ESMO) version, as diagnostic and prognostic criteria of GC recommended indicators of histology, instrumental studies, and her2-status of the tumor [6]. This circumstance underlines the relevance of further studies of microRNAs as candidates for such biomarkers. Some of the microRNAs presented in this work can be selected for verification or combined into new diagnostic or prognostic panels.

## 6. Conclusions

Currently, there are many microRNAs associated with GC development that have been characterized. For a number of them, the relationship with the signaling pathways Wnt/β-catenin, PI3K/AKT/mTOR, RAS/RAF/EKK/MAPK, NF-kB, TGF-β, and JAK/STAT was traced. MicroRNAs can act on multiple target genes, or genes associated with multiple pathways, and function in a signaling network format. Thus, microRNAs are involved in many cellular processes, such as proliferation, metastasis, and apoptosis. Some microRNAs can have a multidirectional effect on cellular processes.

As a result of published data analysis, it was found that microRNAs associated with metastasis most often interact with the Wnt/β-catenin pathway or affect genes that activate EMT when interacting with other pathways. MicroRNAs associated with chemoresistance, in most cases, act on regulators of apoptosis and are associated with the PI3K/AKT/mTOR pathway. 

MicroRNAs have significant potential as markers for the detection and GC monitoring. However, at present, microRNA-based biomarkers are not ready for use in clinical practice. Large-scale studies are required to prove strong associations of some microRNAs with GC and to differentiate the levels of microRNA expression in GC and other gastric pathologies (as well as other nosologies).

## Figures and Tables

**Table 1:** The microRNAs involved in GC metastasis, their direct targets and regulated signaling pathways.

Signal Pathway	MicroRNA	Target	Role in GC Metastasis	Materials	Reference
Wnt/β-catenin	miR-18a miR-19a	*SMAD2*	Overexpression of miR-18a was associated with lymph node metastasis and the distant metastases development. Overexpression of miR-19a was associated with lymph node metastasis.	GC tissues	[49]
	miR-30c-5p	*MTA1*	Decreased expression level of miR-30c-5p was associated with lymph nodes metastasis.	GC tissues	[59]
	miR-33a	*SNAI2*	Overexpression of miR-33a inhibited metastasis and tumor growth in vivo.	GC tissues, cell lines and nude mice	[84]
	miR-93 miR-106a	*TIMP2*	Overexpression of miR-106a and miR-93 was associated with lymph node metastasis and vascular invasion.	FFPE samples, cell lines and GC tissues	[27,64]
	miR-140-5p	*WNT1*	Decreased expression level of miR-140-5p was associated with lymph nodes metastasis.	GC tissues	[85]
	miR-142-5p	*CYR61*	Downregulation of miR-142-5p was associated with the metastases development, relapse and poor prognosis.	GC tissues	[86]
	miR-143 miR-145	*MYO6*	miR-143, miR-145 inhibit EMT and metastasis.	GC tissues, cell lines and nude mice	[60]
	miR-188-5p	*PTEN*	Overexpression of miR-188-5p was associated with lymph nodes metastasis and the distant metastases development.	GC tissues	[48]
	miR-204	*SNAIL*	Decreased expression level of miR-204 was associated with the metastases development.	GC tissues, cell lines and nude mice	[63]
	miR-302b miR-520d-3p	*EPHA2*	Downregulation of miR-302b correlated with the depth of tumor invasion, lymph nodes metastasis, and later stage of GC. Downregulation of miR-520d-3p correlated with increased tumor invasion, lymph node metastasis, and clinical stage.	GC tissues, cell lines and nude mice	[29,30,66]
	miR-330-3p	*PRRX1*	Negative correlation between expression level of miR-330-3p and tumor size, TNM stage, and the degree of lymph nodes metastasis	GC tissues	[87]
	miR-361-5p	Indirectly affects *SNAIL*, *E-cadherin*, *β-catenin*	Decreased expression level of miR-361-5p correlated with lymph node metastasis and the distant metastases development.	GC tissues, cell lines and nude mice	[88]
	miR-516a-3p	*SULF1*	Decreased expression level of miR-516a-3p was associated with the spread of metastases in the peritoneum.	Nude mice	[62]
	miR-520f-3p	*SOX9*	Decreased expression of miR-520f-3p correlated with the depth of tumor invasion, metastases development, and poor prognosis in GC patients.	GC tissues	[89]
	miR-1225-5p	*IRS1*	Decreased expression level of miR-1225-5p correlated with the depth of tumor invasion, lymph node metastasis, and distant metastases.	GC tissues, cell lines and nude mice	[90]
PI3K/AKT/mTOR	miR-28	*PTEN*	MiR-28 knockdown inhibited the proliferative and invasive properties of GC cells.	Cell lines	[71]
	miR-142-5p	*PIK3CA*	Overexpression of miR-142-5p inhibited cell proliferation and tumor growth and metastasis.	GC tissues, cell lines and nude mice	[91]
	miR-197	*MTDH*	Revealed a negative correlation between the expression level of miR-197 and the size of the tumor, the depth of invasion and lymph nodes metastasis.	GC tissues	[72]
	miR-340	*SPP1*	Overexpression of miR-340 induced apoptosis, inhibited proliferation, invasion, in vitro migration, and in vivo tumor growth.	Cell lines and nude mice	[67]
	miR-379	*FAK*	miR-379 was associated with lymph nodes metastasis.	GC tissues	[68]
	miR-498	*BMI-1*	Decreased expression level of miR-498 was associated with lymph node metastasis and poor prognosis in GC patients.	GC tissues	[73]
	miR-520a-3p	*WEE1*	Induced expression of miR-520a-3p decreased tumor size.	Cell lines and nude mice	[69]
	miR-1254	*SMURF1*	Decreased expression level of miR-1254 was associated with an increase in tumor size and the degree of lymph nodes metastasis.	GC tissues, cell lines and nude mice	[70]
NF-κB	miR-9	*D1*, *ETS1*, *NF-κB1*	Decreased expression level of miR-9 was associated with lymph nodes metastasis and the distant metastases development.	GC tissues, cell lines and nude mice	[74,92]
	miR-216a-3p	*RUNX1*	Increased miR-216 expression correlated with lymph node metastasis.	GC tissues	[44]
	miR-508-3p	*NFKB1*	Overexpression of miR-508-3p inhibited the invasive properties of GC cells in vitro. In addition, miR-508-3p suppressed the level of MMP9 expression.	Cell lines and nude mice	[75]
NF-kB; Wnt/β-catenin	miR-7	*RELA, IGF1R*	MiR-7 transfection inhibits distant metastases in vivo.	GC tissues, cell lines and nude mice	[37,38]
	miR-146a	*IRAK1, EGFR*	Decreased expression level of miR-146a was associated with lymph node metastases.	GC tissues	[93]
JAK/STAT	miR-216a	*JAK2*	Decreased expression level of miR-216 correlated with lymph node metastasis.	GC tissues and cell lines	[42]
NF-κB; JAK/STAT	miR-1224	*FAK*	Decreased expression level of miR-1224 was associated with metastasis to lymph nodes in intestinal type of GC.	GC tissues and FFPE tissues	[58]
RHOA-ROCK	miR-31	*RHOA*	Decreased expression level of miR-31 was associated with lymph nodes metastasis. It was shown that miR-31 inhibited the development of distant metastases.	GC tissues, cell lines and nude mice	[94]
RAS/RAF/EKK/MAPK	miR-302b	*CDK2*	Decreased expression level of miR-302b was associated with lymph node metastasis and advanced GC.	GC tissue	[28]
	miR-585	*MAPK1*	Decreased expression level of miR-585 was associated with tumor invasion, lymph node metastasis, and advanced GC.	GC tissues, cell lines and nude mice	[95]
PI3K/AKT/mTOR; RAS/RAF/EKK/MAPK; JAK/STAT and Notch	miR-21 miR-25 miR-221/222 miR-374b-5p miR-590-5p	*RECK*	Overexpression of these microRNAs was associated with lymph nodes metastasis.	GC tissues and cell lines	[80,81,82,83]

**Table 2:** The microRNAs and processes associated with GC chemoresistance.

Signaling Pathway	Process		
	Apoptosis	Apoptosis; Outflow of Drugs from the Cell	Autophagy
PI3K/AKT/mTOR	miR-17-5p miR-21 miR-106a miR-147 miR-193-3p miR-198 miR-375 miR-495 miR-501 miR-4295	miR-20a miR-126 miR-567	miR-148a miR-361-5p
RAS/RAF/ERK/MAPK	miR-135b miR-206 miR-939	miR-20a	
EMT (Wnt/β-catenin)	miR-200a miR-200c miR-204 miR-421 miR-574-3p	miR-34a	
NF-κB	miR-145 miR-146a miR-362-5p	miR-20a	

**Table 3:** MicroRNAs as potential GC biomarkers.

MicroRNA	Characteristics of microRNAs as Candidates for GC Markers	Material/Method	Reference
miR-9	Lymph nodes metastasis (*p* < 0.001) and distant metastases (*p* = 0.022).	GC tissue samples	[92]
miR-19b miR-106a	Diagnostics: AUC = 0.814, sensitivity = 95%, and specificity = 90%.	Circulating exosomal microRNAs	[124]
miR-21 miR-106a	Difference in expression in gastric and non-cancerous cancers (*p* < 0.001).	Gastric juice	[125]
miR-23a miR-135	Diagnostics: miR-23a specificity, sensitivity, and AUC are 67.95, 87.50, and 0.805%, respectively, for miR-135 at 73.08, 82.50, and 0.824%, respectively.	Serum	[126]
miR-24 miR-101	OS (*p* < 0.01).	GC tissue samples	[127]
miR-29c	Decreased expression level in tumor tissue compared to unaffected mucosa (*p* < 0.0001).	GC tissue samples	[128]
miR-30c-5p	Lymph node metastasis (*p* = 0.014).	GC tissue samples	[59]
miR-93	Lymph node metastasis (*p* < 0.01).	GC tissue samples	[64]
miR-101-3p	When distinguishing atrophic gastritis and gastric cancer: AUC = 0.8749, sensitivity = 72.09%, and specificity = 86.49%.	Serum	[129]
miR-106	Sensitivity = 0.71, specificity = 0.82, and AUC = 0.80.	Serum, tissue, plasma and gastric juice	[130]
miR-106a	Lymph node metastasis (*p* = 0.002), vascular invasion (*p* = 0.017), and the depth of tumor invasion (*p* = 0.009).	GC tissue samples	[27]
miR-107	The expression level was increased in adenoma with high-grade dysplasia *p* = 0.006 and in the early stages of GC *p* = 0.03.	Microarrays, validation by RT-PCR	[131]
miR-129	Significantly lower levels of miR-129-1-3p (*p* = 0.007), miR-129-2-3p (*p* = 0.003), and a combination of two microRNAs (*p* = 0.003) in GC patients compared to patients with benign stomach diseases.	In gastric juice	[125]
miR-140-5p	Lymph node metastasis (*p* = 0.018); survival—PFS, *p* < 0.05, OS, *p* < 0.05).	GC tissue samples	[85]
miR-155 miR-223	Atrophic gastritis (*p* < 0.0001); gastric cancer (*p* < 0.05).	GC and AG tissue samples	[132]
miR-181a	Lymph node metastasis (*p* = 0.0124); distant metastases (*p* = 0.0376).	GC tissue samples	[133]
miR-181d	Lymph node metastasis (*p* < 0.05) and overall survival (*p* = 0.001).	GC tissue samples	[134]
miR-196a	Survival: (*p* = 0.032, HR = 3.057, 95% CI = 1.1–8.495); Diagnostics: AUC = 0.864, sensitivity 69.5%, specificity 97.6%; metastasis *p* < 0.001.	Plasma	[135]
miR-196b	Survival: *p* = 0.042, HR = 2.914, 95% CI = 1.036–8.174; diagnostics: AUC = 0.811, sensitivity 62.2%, and specificity 96.1%.	Plasma	[135]
miR-197	Depth of invasion (*p* = 0.005), lymph node metastases (*p* = 0.004).	GC tissue samples	[72]
miR-200c	Had a prognostic value and moderately diagnostic. Expression data were inconsistent.	Meta-analysis	[136]
miR-302b	Lymph node metastases (*p* = 0.003), OS (HR = 1.86; 95% CI, 1.11–3.14; *p* = 0.021).	GC tissue samples	[29]
miR-330-3p	Lymph node metastasis (*p* < 0.001).	Serum, GC tissue samples	[87]
miR-361-5p	Lymph node metastasis, distant metastases development (*p* < 0.001).	GC tissue samples	[88]
miR-376a	Metastasis to regional lymph nodes (*p* = 0.02) and poor prognosis (*p* = 0.02).	Tissues and cell lines of GC	[137]
miR-379	Lymph node metastasis (*p* < 0.001), OS (*p* = 0.0007), and PHS (*p* = 0.0002).	GC tissue samples	[68]
miR-421	Difference in expression among patients with benign and malignant gastric diseases (*p* < 0.001).	Gastric juice	[125]
	OS (*p* = 0.016, HR 2.586, 95% CI 1.194–5.599) and RFS (*p* = 0.014, HR 2.465, 95% CI 1.201–5.060).	GC tissue samples	[109]
	Early stages. Sensitivity 96.67; specificity 95.56; AUC 0.981 (0.942–0.997); *p* <0.0001.	Serum	[138]
miR-484	Lymph node metastasis (*p* = 0.015), distant metastases development (*p* = 0.005), stage of the disease (*p* = 0.002), and degree of differentiation (*p* = 0.006).	GC tissue samples	[139]
miR-519a	Lymph node metastasis, degree of differentiation, and stage of the disease (*p* < 0.05); OS (*p* = 0.002).	GC tissue samples	[140]
miR-520a-3p	Depth of tumor invasion (*p* < 0.001) and stage of the disease (*p* < 0.05).	GC tissue samples	[141]
miR-552	Lymph node metastasis (*p* = 0.018) and OS (*p* = 0.011), HR = 5.657, 95% CI 1.619–19.761.	GC tissue samples	[142]
miR-585	Depth of tumor invasion (*p* < 0.010), lymph node metastasis (*p* < 0.002).	GC tissue samples	[95]
miR-601	Invasion, lymph node metastasis, and the distant metastases development (*p* < 0.05); OS (*p* = 0.001).	GC tissue samples	[143]
miR-1225-5p	Depth of tumor invasion (*p* = 0.016), spread of metastases to lymph nodes (*p* = 0.002), and development of distant metastases (*p* = 0.01).	GC tissue samples	[90]
miR-1236-3p	Lymph node metastasis (*p* = 0.005), disease stage (*p* = 0.001), and degree of differentiation (*p* = 0.001).	GC tissue samples	[144]
Potential markers of response to chemotherapy			
miR-27b miR-508-5p	Response to chemotherapy (*p* = 0.02 and *p* = 0.04, respectively).	GC tissue samples	[145]
miR-939	Potential marker of sensitivity to chemotherapy; AUC = 0.777, *p* < 0.001.	GC tissue samples	[105]
MicroRNA panels			
miR-7-2 miR-9-3 miR-548o miR-1255a miR-3687	Patient survival AUC = 0.9 (HR, 2.840; 95% CI, 1.937–4.162; *p* < 0.01).	TCGA database	[146]
miR-143-3p miR-146a miR-451a miR-501-3p	Predicting the development of lymph node metastases in GC; AUC = 0.822 (95% CI, 0.758 to 0.875), specificity = 87.78%, and sensitivity = 63.33%.	Serum	[147]
miR-22-5p miR-132-3p miR-200a-3p miR-485-3p miR-2965p	Suggested for diagnostics of GC AUC= 0.724.	Serum	[148]
miR-10b-5p miR-20a-3p miR-132-3p miR-185-5p miR-195-5p miR-296-5p	Suggested for diagnostics of GC AUC = 0.702.	Serum	[149]

AUC—area under the curve; PFS—progression-free survival; RFS—relapse-free survival; OS—overall survival; HR—hazard ratio; CI—confidence interval; RR—relative risk.
